# Economic evaluation of breast MRI in screening - a systematic review and basic approach to cost-effectiveness analyses

**DOI:** 10.3389/fonc.2023.1292268

**Published:** 2023-12-07

**Authors:** Fabian Tollens, Pascal A.T. Baltzer, Matthias F. Froelich, Clemens G. Kaiser

**Affiliations:** ^1^ Department of Radiology and Nuclear Medicine, University Medical Centre Mannheim, Medical Faculty Mannheim, University of Heidelberg, Mannheim, Germany; ^2^ Department of Biomedical Imaging and Image-Guided Therapy, Vienna General Hospital, Medical University of Vienna, Vienna, Austria

**Keywords:** breast cancer screening, breast MRI, abbreviated breast MRI, MR-mammography, cost-effectiveness analysis, economic evaluation

## Abstract

**Background:**

Economic evaluations have become an accepted methodology for decision makers to allocate resources in healthcare systems. Particularly in screening, where short-term costs are associated with long-term benefits, and adverse effects of screening intermingle, cost-effectiveness analyses provide a means to estimate the economic value of screening.

**Purpose:**

To introduce the methodology of economic evaluations and to review the existing evidence on cost-effectiveness of MR-based breast cancer screening.

**Materials and methods:**

The various concepts and techniques of economic evaluations critical to the interpretation of cost-effectiveness analyses are briefly introduced. In a systematic review of the literature, economic evaluations from the years 2000-2022 are reviewed.

**Results:**

Despite a considerable heterogeneity in the reported input variables, outcome categories and methodological approaches, cost-effectiveness analyses report favorably on the economic value of breast MRI screening for different risk groups, including both short- and long-term costs and outcomes.

**Conclusion:**

Economic evaluations indicate a strongly favorable economic value of breast MRI screening for women at high risk and for women with dense breast tissue.

## Introduction

Breast cancer is the most common cancer and the leading cause of cancer-related death in women worldwide with an estimated 2.3 million incident cases and 685,000 deaths in 2020, despite significant advances in therapeutic options and widespread screening programs (1, 2). Diagnosed at an early stage, localized breast cancer, much like colorectal cancer, is associated with excellent 5-year survival rates of approximately 99% (3). Due to the lack of symptoms in an early stage, screening for breast cancer is particularly promising and relevant.

For conventional screening programs, reductions in breast cancer mortality have been demonstrated (4–6), even though the positive results have been a matter of scientific discussion: some authors critically remark the high number of false positive cases (7, 8) and the imperfect sensitivity of mammography. Other authors derive benefits in survival predominantly from advances in breast cancer therapy and an effect of overdiagnosis (9). On top, the risk of radiation-induced cancers must be considered (10).

Among the various modalities applied in breast imaging, breast MRI is accepted to have the highest sensitivity in detecting breast cancer independent from breast density (11). Concerns on specificity and high costs, among other reasons, have averted breast MRI from taking a prominent role in screening.

The most recent multi-center studies have demonstrated that breast MRI does not suffer from reduced specificity compared to conventional mammography (12–14). However, reader experience, quality assurance and continuous monitoring are considered prerequisites for optimizing the diagnostic performance of breast MRI.

While evidence on the superior diagnostic performance of breast MRI in screening women at high risk has been available for several years (15–17), prospective multi-centric data for women with dense breasts have become available only recently and have confirmed superior sensitivity of 95.2% - 95.7% and reduced interval cancer rates of MRI-based screening compared to conventional approaches (18–20). Specificity increased in subsequent screening rounds (incidence rounds) as compared to the first screening round (prevalence round). In general, MRI-detected cancers were smaller than tumors detected by conventional mammography (21), and biologically aggressive cancers are more likely to be detected by MRI (22).

Besides requirements of efficacy, safety, and acceptance of screening, costs and potential benefits of screening programs need to be economically balanced (23). Innovative screening programs and expensive diagnostic tests are required to not only provide superior efficacy but also favorable economic effects (24). As a consequence, both short- and long-term costs and outcomes of screening are increasingly assessed by economic evaluations in order to capture their economic potential and to direct healthcare resource allocation accordingly. Cost-effectiveness analyses have evolved as an established framework for estimating economic value of innovative screening measures based on economic modeling and represent a prerequisite to establish funding by health insurance funds in various healthcare systems (25).

There are various methodological approaches with different outcome categories reported, hampering comparability of the findings and misleading economically inexperienced readers (26, 27).

However, for the various diagnostic modalities in breast imaging, each with different diagnostic potential and financial burden, cost-effectiveness analyses are particularly valuable and may help identify the most efficient medical care for each risk group.

Firstly, we introduce various methodologies of economic evaluations, explain the different approaches of outcome measurement and aim at developing a conceptual understanding of economic evaluations. Secondly, in the systematic review of the literature, the latest available evidence on cost-effectiveness of MRI-based breast cancer screening is discussed and evaluated.

## A brief guide to economic evaluations

In health economics, evaluations are conducted to systematically compare different diagnostic or therapeutic strategies, e.g. the standard of care versus an innovative technique. Not only the costs of medical interventions can be considered but a certain “value” can be assigned to the outcomes. Capturing the value of diagnostic radiology can be challenging since diagnostic techniques only indirectly affect health care outcomes (28).

Empirical studies on the economic value of long-term patient journeys and health services administration are often not feasible due to associated costs and time constraints, and controlled experiments may be difficult to implement due to ethical and medical concerns. To overcome these limitations, the contemporary methodology is based on economic modeling and theoretical decision analysis that are applied to simulate the alternating diagnostic or therapeutic pathways, including all relevant medical costs and associated outcomes (24).

### Measurement of costs

Various perspectives can be assumed to estimate costs, e.g. the perspective of the healthcare system, society or healthcare provider (29). Depending on the perspective, different costs have to be considered, e.g. direct medical costs including costs of treatment and personnel, indirect medical costs including transportation costs and intangible costs including non-monetary factors such as quality of life. For example, absence from work due to disease may result in productivity losses on the level of the economy that can be expressed in monetary terms.

### Measurement of outcomes

There are various outcome categories applicable in economic modeling: Outcomes can be measured in monetary terms, in natural units such as mmHg blood pressure reduction, or life years gained. However, the heterogeneity of outcomes intrinsically limits comparability and transferability of the consecutive results.

Considering changes in life expectancy (life years gained) allows comparisons across various conditions. However, differences in quality of life (QoL) are neglected, e.g. due to side effects of therapies. Therefore, quality-adjusted life years (QALYs) have evolved as a reference standard as well as generic means of outcome measurement. QALYs include both the quantity as well as quality of life time, obtainable by multiplying life time with quality of life (29). This way, generic outcomes can be compared between different diseases and therapies. Hence, QALYs have become the gold standard in measuring health care outcomes in cost-effectiveness- or cost-utility analyses.

When estimating outcomes in cost-effectiveness modeling, a practical concern is the availability of input variables to construct valid economic models. Data on quality of life are still scarce for many conditions, and the methodological variability of valuing utilities may limit their validity (30). It may be difficult to quantify the quality of life of any health state. Literature on quality of life is growing, and an increasing number of prospective study designs include QoL-measurements as well.

### Types of economic evaluations

There are various types of economic evaluations (29): In cost-benefit analyses, outcomes are expressed in monetary terms. Cost-effectiveness analyses try to relate costs to natural outcomes such as reduction of cholesterol levels or life years gained. Cost-utility analyses use quality-adjusted life years as generic outcomes and are considered a reference standard as they enable comparisons across conditions based on a common denominator, i.e. QALYs. In the literature, the terms cost-effectiveness analysis and cost-utility analysis are often used interchangeably.

### Decision analysis and Markov-Models

For an economic evaluation based on modeling, a decision tree is necessary including the therapeutic or diagnostic strategies and representing all possible outcomes. Each branch of the decision tree is assigned a predefined probability.

For each branch of the decision tree, a Markov-Model is used as a state-transition model to simulate costs and effects over a predefined time horizon (31). The Markov states are mutually exclusive and collectively exhaustive which means they represent all possible and necessary disease states. Patients may freely transition from one Markov state to another after each cycle, as they receive treatments or experience changes in health states. A Markov model is “memoryless”, which means that the transition probabilities assigned to each Markov state do not depend on the history of prior Markov states but only on the current disease state. A fixed cycle length is chosen depending on the modeled disease entity. Associated costs and outcomes are assigned to each Markov state.

Economic modeling of breast cancer screening incorporates not only the costs and outcomes of screening tests and follow-ups, but also stage-dependent costs of therapeutic pathways and consecutive reductions in quality of life. True positive and true negative results are included as well as false negative and false positive findings.

**Figure 1:**
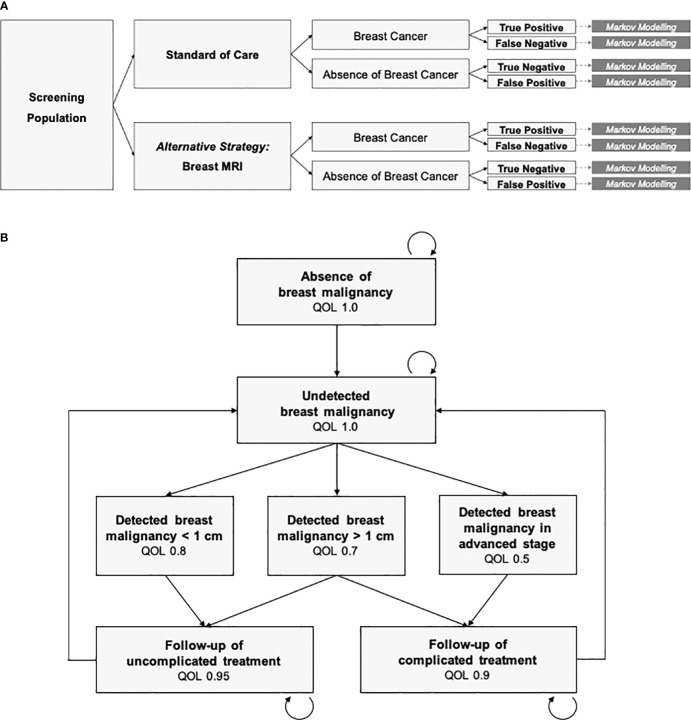
Decision analysis and economic modeling. **(A)** Decision tree including the diagnostic strategies (standard of care vs. breast MRI), ground truth (breast cancer vs. absence of breast cancer) and the diagnostic outcomes. Markov Modeling is conducted for each branch of the decision tree. **(B)** The Markov Model is defined by the mutually exclusive and collectively exhaustive Markov states, cycle length, transition probabilities, and the costs and quality of life (QOL) assigned to each state. Mortality is included in any state.

An exemplary decision tree and Markov model to simulate breast cancer screening is depicted in **Figure 1**. All possible diagnostic outcomes are included: True positive, false negative, true negative and false positive findings. The set of Markov states needs to be differentiated enough to represent the variety of all possible disease states. However, it also needs to be simple enough to be based on valid estimates of input variables, in order to prevent from getting lost in assumptions on subgroups and pre-conditions. From a practical point of view, identification of valid point estimates for the input variables is crucial and depends on the quality of the underlying evidence.

### Microsimulation models

While Markov models offer a means to simulate cohorts of patients based on cohort averages, and the simulation is memoryless by its classical definition (“Markov assumption”), microsimulation models represent an alternative technique.

Microsimulation is used to model individual patients’ histories that are characterized by predefined variables and sets of rules (32), which is computationally more demanding. Due to their higher complexity, microsimulation models usually require a more thorough design, detailed and epidemiological input data as well as model validation. For instance, Microsimulation Screening Analysis (MISCAN) models have been designed to examine various cancer entities (33).

### Incremental cost-effectiveness ratios and willingness to pay

When comparing alternative health care strategies, e.g. an innovative technique versus the established standard of care, additional costs per certain outcome are calculated and expressed as the ICER:


$$\begin{array}{l} ICER\;=\;\frac{incremental\;cost}{incremental\;effectiveness}\\ =\frac{\;cost\;alternative\;therapy\;-\;cost\;standard\;therapy\;}{\;effectiveness\;alternative\;therapy\;-\;effectiveness\;standard\;therapy} \end{array}$$

The ICER as a measure of cost-effectiveness can be used by decision makers to direct resource allocation in healthcare systems. The adoption of a new medical procedure is favored when the ICER falls below the WTP-threshold. There is a substantial global heterogeneity in the value of health and the resulting WTP for medical services. Thresholds differ between countries, healthcare systems and individual contexts, and depend on various factors such as reimbursement schemes, availability of services and resources, individual preferences and cultural factors. For instance, developing countries may not be comparable to developed countries, and there is substantial heterogeneity even within Western industrialized countries.

In the United States, a WTP-threshold between US-dollar (USD) 50,000 and USD 200,000 per QALY gained has been discussed, whereas a threshold of £ 20,000 - 30,000 has been adopted for the United Kingdom (34, 35). The world health organization has proposed to use the gross domestic product (GDP) per capita as a threshold that indicates high cost-effectiveness and 3 x the GDP indicating cost-effectiveness (36).

**Figure 2:**
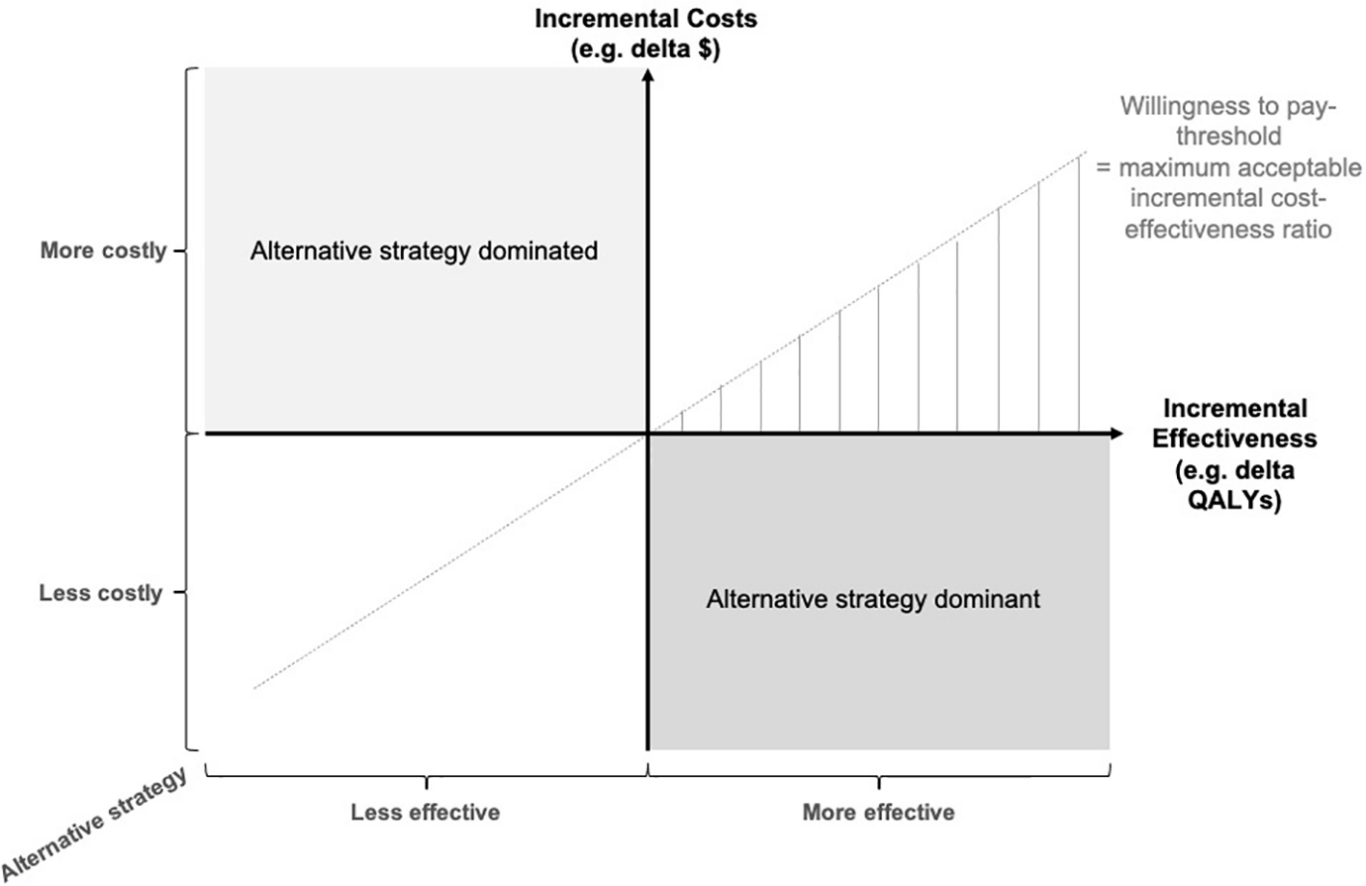
Cost-effectiveness plane. The incremental costs (e.g. in $) and incremental effects (e.g. in quality-adjusted life years, QALYs) of the alternative strategy, e.g. breast MRI, are computed to locate the strategy on the cost-effectiveness plane. In case it is more costly and less effective than the standard of care, the alternative strategy is dominated. In case of smaller costs and additional effectiveness, the alternative strategy is dominant. In the case of more effectiveness but more costs, the incremental cost-effectiveness ratio (ICER) has to be smaller than the willingness to pay (WTP) - threshold to be economically preferable.

In a cost-effectiveness plane, incremental costs and effects of various strategies are displayed. In case a strategy achieves superior outcomes at reduced costs, the strategy is preferred (dominant strategy). In case a strategy achieves superior outcomes, but is associated with increased costs, the ICER is reflected by the slope of the line (**Figure 2**).

### Sensitivity analyses

Sensitivity analyses are conducted in order to address variability of the input parameters and uncertainty in the model design, and to estimate robustness of model outcomes (37). Input variables are point estimates and often represent the population average. In a deterministic sensitivity analysis, an input variable is varied within a predefined range and the model outcomes are computed. For instance, a range of costs per breast MRI has been reported for different health care systems and providers, and depending on reader experience, sensitivity and specificity of breast MRI may vary. These uncertainties can be addressed by simulating outcomes for a range of possible input values.

However, most variables can not only be expressed by a population average, but follow a probability distribution in the respective population. Therefore, a Monte Carlo simulation is conducted by randomly assuming values from the probability distribution of every variable in the model simultaneously. In a probabilistic sensitivity analysis, the resulting cost-effectiveness is simulated for a significant number of iterations, e.g. 30,000 iterations.

### Quality assurance and checklists

In order to maintain a high standard of quality, extensive recommendations on the methodological conduct of cost-effectiveness analyses have been defined (38). Checklists are available to evaluate adherence to these recommendations. For instance, the Consolidated Health Economic Evaluation Reporting Standards (CHEERS) Statement (39) is widely applied to ensure appropriate reporting (**Supplementary Table S1**).

## Materials and methods: systematic review

**Figure 3:**
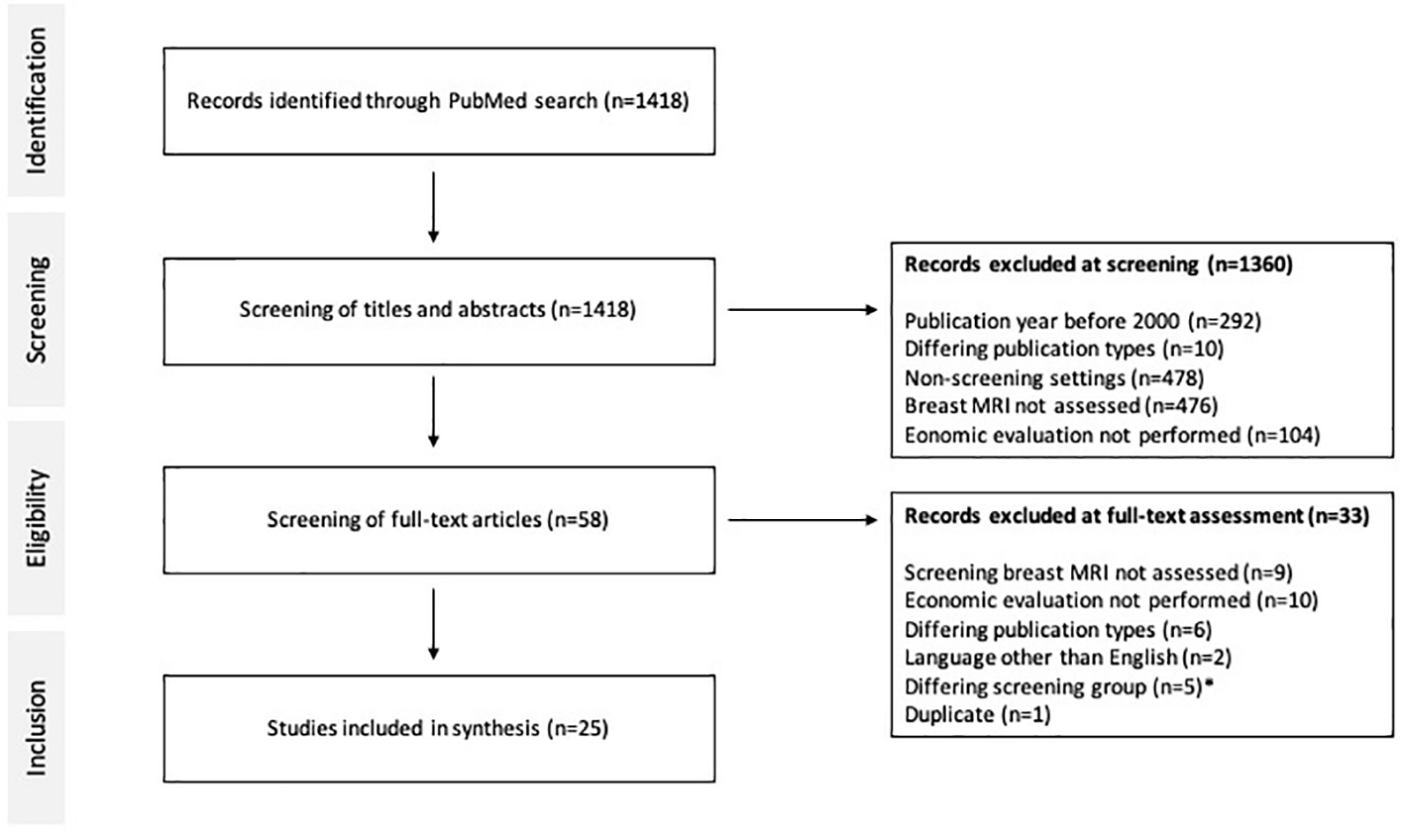
PRISMA diagram and literature search. Economic evaluations between 2000 and 2022 on breast MRI in screening of breast cancer were included. * Exceptional patient subgroups such as dialysis patients and childhood survivors of cancer or lymphoma were excluded.

In a systematic review of economic evaluations on breast MRI screening, the PubMed database was scanned for literature between January 1, 2000 and November 25th, 2022 (**Figure 3**). Key-words included “breast MR*”, “breast cancer screening”, “MR-mammography”, “magnetic resonance imaging screening”, and “cost effective”, “cost-benefit”, “economic evaluation”, “cost-utility”, or “cost”. Economic evaluations including cost-effectiveness, cost-utility and cost-benefit analyses on breast cancer screening that applied screening MRI were included into analysis.

## Results

In total, 1418 studies were identified on PubMed, 1360 were excluded during screening of abstracts and 33 were excluded during full-text analysis (**Figure 3**). Finally, 25 articles on economics of breast MRI screening of different risk groups were included into further analysis. The CHEERS checklist was used to assess the quality of the included studies (**Supplementary Table S2**). Various indications of MRI-based screening have been established in the past years (**Table 1**).

**Table 1:** International recommendations for breast MRI in breast cancer screening stratified for risk groups.

Risk groups	ACS (2007 and 2015) (40, 41)	ACR (2017, 2018 and 2021) (42–44)	EUSOMA (2010) (45)	EUSOBI (2016 and 2022) (46, 47)
**High risk**	Annual MRI screening for: - *BRCA* mutation carriers and first-degree relatives - women with lifetime risk > 20-25% - Women with Li-Fraumeni, Cowden, Bannayan-Riley-Ruvalcaba syndromes and first-degree relatives (expert consensus) - women who had mantle radiotherapy under 30 years of age (expert consensus)	Annual MRI screening beginning at age 25-30 for: - women with genetics-based increased risk and untested first-degree relatives - women with history of chest radiation with cumulative dose of > 10 Gy before age 30 - lifetime risk > 20%	Annual screening for: - *BRCA1*, *BRCA2*, and *TP53* mutation carriers and first-degree relatives - women with lifetime risk > 20-30% and unclear mutation status (DoR-B) - women who had mantle radiotherapy under 30 years of age (DoR-B)	MRI-based screening according to national or international guidelines is favored
**Intermediate risk**	Insufficient evidence for - Women with 15-20% lifetime risk - lobular intraepithelial neoplasia, atypical ductal hyperplasia - heterogeneously or extremely dense breasts - personal history of breast cancer including DCIS	- Annual MRI for women with personal history of breast cancer and dense breast tissue or diagnosed before age 50 - MRI should be considered for women with history of breast cancer, LCIS or atypia on prior biopsy	n/a	Women with extremely dense breasts aged 50 - 70: - Supplemental screening - preferably by MRI - at least every 4 years, preferably every 2-3 years - MRI can be used as a stand-alone technique
**average risk**	women at lifetime risk< 15%: not recommended	No recommendation due to Insufficient evidence	n/a	n/a

n/a, Not applicable.

### Cost-effectiveness of breast MRI in high-risk screening

The superior diagnostic performance of breast MRI in women at high risk of breast cancer has repeatedly been demonstrated over the past three decades. The most recent prospective multi-center trials confirm a superior sensitivity of 90 - 93% and a specificity of 89 - 98% in women at high risk of breast cancer, whereas mammography achieved a sensitivity of 33 - 50% and a specificity of 97 - 99% (15–17).

Based on the broad evidence available for several years, a number of economic evaluations have assessed the cost-effectiveness of breast MRI in the high-risk group (**Table 2**) (42–44, 48–60). The associated cost per breast MRI examination decreased over the previous years. For instance, in 2006 Plevritis et al. assumed a cost of USD 1,038, whereas contemporary analyses assume costs of USD 314 (48). They demonstrated that screening with mammography and MRI could be cost-effective especially for middle-aged breast cancer gene 1 (BRCA 1) mutation carriers vs. BRCA 2 mutation carriers with dense breast tissue at an ICER of USD 55,420 vs. USD 98,454 per QALY, respectively (48). In Canadian women with mutations in the BRCA 1 or 2 gene, alternating screening with conventional mammography and breast MRI every six months compared to annual mammography alone was cost-effective with an ICER of Canadian dollars (CAD) 50,900 per QALY gained (54).

**Table 2:** Economic evaluations on MRI-based breast cancer screening of women at high risk.

Study	Study population	Economic model; perspective	Comparators	Cost of MRI	Outcome measures	WTP-threshold
Plevritis et al. (48)	*BRCA 1* and *BRCA 2* mutation carriers	Monte Carlo (Microsimulation) Model, U.S. healthcare system	Annual mammography, annual supplemental breast MRI at different age ranges	USD 1,038 (2005)	Costs in USD, QALYs, LYs, ACER (cost/QALY), ICER (cost/QALY)	USD 100,000/QALY gained
Griebsch et al. (49)	Family history of breast cancer or *BRCA 1*, *BRCA 2* or *TP53* mutation carriers or 50% risk of inherited mutation	Markov Chain simulation, NHS, UK healthcare system	Annual breast MRI, mammography, or combination of both	£ 250 - 299 (2003/2004)	Costs in £, costs per cancer detected	n/a
Norman et al. (50)	*BRCA 1* mutation carriers	Markov Model, National Health Service, United Kingdom	No screening, annual mammography, breast MRI or combination of both, for different age groups	£ 224 (2006)	Costs in £, QALYs, ICER (cost/QALY)	£ 20,000/QALY gained
Moore et al. (51)	Women with 15% cumulative lifetime risk or higher	Markov Model, U.S. healthcare system	Annual mammography or breast MRI	USD 966 (2006)	Costs in USD, QALYs, ICER (cost/QALY)	USD 50,000 - 200,000/QALY gained
Taneja et al. (42)	*BRCA 1* and *BRCA 2* mutation carriers, other high-risk characteristics (lifetime risk ≥20%)	Decision analytic Model, U.S. healthcare system	Annual breast MRI, mammography and combination of both	USD 1,038 initially, USD 787 for follow-up screening (2005)	Costs in CAD, life expectancy, QALYs, ICER (cost/QALY)	n/a
Lee et al. (43)	*BRCA 1* mutation carriers	Markov Model, societal perspective	Annual mammography, breast MRI or combination of both	USD 577 (2007)	Costs in USD, LYs, QALYs, ICER (cost/QALY)	USD 100,000 and 50,000/QALY gained
Grann et al. (44)	*BRCA 1* and *BRCA 2* mutation carriers	Markov Model, U.S. healthcare system	Annual mammography with and without breast MRI	USD 1,219 initially, USD 940 for short interval follow-up (2009)	Costs in USD, QALYs, ICER (cost/QALY)	n/a
Cott Chubiz et al. (52)	*BRCA 1* and *BRCA 2* mutation carriers	Markov Monte Carlo Model, U.S. healthcare system	Annual mammography and breast MRI starting at different ages	USD 619 (2010)	Costs in USD, QALYs, ICER (cost/QALY)	n/a
De Bock et al. (53)	*BRCA 1* and *BRCA 2* mutation carriers	Microsimulation Model, Dutch and UK healthcare system	Different combinations of mammography and MRI	€ 227 (2013) or £ 220 (2007)	Costs in € or £, LYG, incremental costs per LYG	€ 20,000/LYG and £ 25,000/LYG
Pataky et al. (54)	*BRCA 1* and *BRCA 2* mutation carriers	Markov Model, Canadian healthcare system	Annual mammography, annual supplemental breast MRI	CAD 277 (2008)	Costs in CAD, QALYs, ACER (cost/QALY), ICER (cost/QALY)	CAD 100,000 and 50,000/QALY gained
Saadatmand et al. (55)	Women with familial risk	Microsimulation Model, Dutch healthcare system	Mammography, breast MRI, at different intervals	USD 485 (2013)	Costs in USD and €, LYG, average and incremental costs/LYG	n/a
Ahern et al. (56)	Women at high risk, different life-time risk thresholds	Microsimulation Model, U.S. healthcare system	Annual or biennial breast MRI and mammography at 6-, 12- or 24-month intervals	USD 728 (2012)	Costs in USD, QALYs, LY, ICER (cost/QALY)	USD 100,000/QALY gained
Obdeijn et al. (57)	*BRCA 1* mutation carriers	Microsimulation model, Dutch screening program	Dutch screening guidelines with MRI and mammography, modified protocol with mammography postponed to age 40	€ 368 (2016)	Costs in €, LYG, incremental costs/LYG	n/a
Phi et al. (58)	*BRCA 1* and *BRCA 2* mutation carriers aged 60-74	Microsimulation model, Dutch screening program	Annual mammography, breast MRI, different combinations and screening intervals for women with dense breasts or all women	€ 168 (2017)	Costs in €, LYG, costs/LYG	€ 20,000/life year gained
Geuzinge et al. (59)	Women with 20% or more familial risk without a known *BRCA1/2* or *TP53* mutation	Microsimulation model, Dutch healthcare system	Annual mammography, breast MRI, with various intervals and age groups	€ 272 (2018)	Costs in €, LY, QALYs, ICER (cost/QALY)	€ 22,000/QALY gained
Kaiser et al. (60)	Women at high risk	Markov Model, U.S. healthcare system	Annual mammography, ultrasound, mammography and ultrasound, breast MRI	USD 385 (2021)	Costs in USD, QALYs, ICER (cost/QALY)	USD 100,000/QALY gained

n/a, Not applicable.

Addressing the impact of specificity on cost-effectiveness of high-risk screening, Kaiser et al. have simulated the ICER for varying levels of specificity in women with high risk of breast cancer based on annual screening intervals (60). Compared to conventional mammography, breast MRI remained cost-effective at a WTP-threshold of USD 100,000 per QALY as long as the specificity did not drop below 86.7%.

Simulating various screening intervals and combinations of breast MRI and conventional mammography for the Dutch healthcare system, Geuzinge et al. found breast MRI in 18-month intervals between the ages of 35 and 60 years to be most cost-effective at an ICER of € 21,380 per QALY gained (59).

In a recent review including economic evaluations from 2006-2019, Li et al. proposed precision screening strategies tailored to age and individual risk from an economical perspective (61). Mammography and additional breast MRI were predominantly cost-effective for BRCA1 mutation carriers in middle-age groups, whereas additional breast MRI was not cost-effective for BRCA 2 mutation carriers.

### Cost-effectiveness of breast MRI in intermediate-risk screening

MRI screening in women with dense breast tissue, i.e. intermediate risk for breast cancer, has recently demonstrated excellent outcomes. In the DENSE trial, women with extremely dense breast tissue were offered supplemental MRI screening in the Netherlands. The cancer detection rate dropped from 16.5 per 1000 examinations in the first round to 5.8 per 1000 in the second screening round (18, 19). At the same time the false positive rate decreased from 79.8 to 26.3 per 1000 examinations.

Kaiser et al. have first demonstrated the favorable economic value of breast MRI as a screening technique in women with extremely dense breast tissue (62) based on the findings from the first round of the DENSE study (**Table 3**). Compared to conventional mammography, they calculated an ICER of USD 8,798 per QALY gained for biennial screening with breast MRI.

**Table 3:** Economic evaluations on MRI-based breast cancer screening of women at intermediate risk due to elevated breast tissue density.

Study	Study population	Economic model; perspective	Comparators	Cost of MRI	Outcome measures	WTP-threshold
Kaiser et al. (60)	Women with extremely dense breast tissue	Markov Model, US healthcare system	Biennial breast MRI or mammography	USD 385 (2021)	Costs in USD, QALYs, ICER (cost/QALY)	USD 100,000/QALY gained
Tollens et al. (63)	Women with heterogeneously and extremely dense breast tissue	Markov Model, US healthcare system	Biennial abbreviated protocol breast MRI or DBT	USD 314 (2021)	Costs in USD, QALYs, ICER (cost/QALY)	USD 100,000/QALY gained
Tollens et al. (63)	Women with extremely dense breast tissue	Markov Model, US healthcare system	Biennial mammography or breast MRI	USD 314 (2021)	Costs in USD, QALYs, ICER (cost/QALY)	USD 100,000/QALY gained
Geuzinge et al. (64)	Women with extremely dense breast tissue	Microsimulation model, Dutch screening program	Breast MRI, mammography, and combinations thereof, different screening intervals	€ 272 (2018)	Costs in €, number of breast cancers, life years gained, breast cancer deaths, overdiagnosis, QALYs, ICER (cost/QALY)	€ 22,000/QALY gained
Wang et al. (65)	Women with heterogeneously and extremely dense breast tissue	Microsimulation model, Dutch screening program	Abbreviated protocol breast MRI, conventional mammography, and combinations thereof, different screening intervals	€ 272 (2019)	Costs in €, breast cancer deaths, LYG, incremental cost/LYG, average cost/LYG	€ 20,000/LY gained
Tollens et al. (66)	Women with dense breast tissue	Markov model, US healthcare system	Biennial breast MRI, full diagnostic protocols vs. abbreviated protocols	USD 314 (2022)	Costs in USD, QALYs, ICER (cost/QALY)	USD 100,000/QALY gained

Considering the shift in diagnostic performance of breast MRI in the second screening round, i.e. increased specificity of breast MRI reported by the DENSE study group, as well as the reduced cancer detection rate of the second screening round (incidence round) compared to the first round (prevalence round), Tollens et al. confirmed the cost-effectiveness of breast MRI in this patient collective with a further refined Markov-Model (63). When the reduced false positive rate and cancer detection rate from the second screening round are projected on subsequent screening rounds, the reported ICER dropped from USD 38,849 to USD 13,493 per QALY. The authors concluded that the reduced false positive findings and reduced associated follow-up costs outweighed the reduced cancer detection rate from an economic perspective.

Long-term outcomes were also simulated by microsimulation modeling (MISCAN) based on the DENSE trial data and estimated cost-effectiveness of screening women with extremely dense breasts (64). Comparing biennial MRI to biennial mammography would save 8.6 additional lives per 1,000 women invited and cost € 22,500 per QALY gained. In this simulation, MRI screening alone every 4 years saved 7.6 additional lives per 1,000 women at a cost of € 11,500 per QALY gained.

Examining the economic potential of abbreviating MRI protocols for breast cancer screening patients of intermediate risk, evidence on diagnostic performance is scarce. Comparing abbreviated breast MRI to digital breast tomosynthesis (DBT) in women with dense breasts and extremely dense breasts, the EA1411 ECOG-ACRIN study determined a cancer detection rate of 11.8 per 1000 examinations for abbreviated breast-MRI (AB-MRI) and 4.8 per 1000 for DBT (20). No interval cancers were observed. Comstock et al. reported similar levels of sensitivity, yet reduced levels of specificity of AB-MRI.

A simulation of long-term costs and outcomes by Tollens et al. (66) based on the data of Comstock et al. confirmed the cost-effectiveness of AB-MRI in screening women of intermediate risk for breast cancer, including increased false positive findings of abbreviated examinations. As long as the cost of AB-MRI did not exceed 82% of the cost of a full protocol examination, AB-MRI should be considered the cost-effective alternative.

In women with heterogeneously and extremely dense breasts, MRI screening with abbreviated protocols was cost-effective across a wide range of plausible costs per examination when compared to DBT (67). When varying the assumed cost per examination, abbreviated breast MRI was cost-effective below USD 593 and cost-saving below USD 241 compared to DBT.

Wang et al. used the SiMRiSc microsimulation model to compare different screening scenarios including conventional mammography and abbreviated breast MRI in screening women with dense breasts in the Netherlands (65). Costs associated with implementation of a screening program, the involution of breast tissue over time, and radiation-induced tumors were incorporated as well. Biennial MRI screening from 50 - 65 years plus mammography from 66 - 74 years for women with extremely dense breasts was identified as the optimal strategy at an ICER of € 18,201 per life year gained (LYG). Other screening scenarios applying more extensive MRI screening, e.g. biennial MRI from 50 - 74 years, achieved even more LYG and smaller interval cancer rates, yet at an ICER above the predefined WTP-threshold of € 20,000 per LYG.

### Cost-effectiveness of breast MRI in average-risk screening

As of today, data on the diagnostic performance of breast MRI in average-risk collectives is limited.

Screening women with average risk of breast cancer with supplemental breast MRI, including women with dense breast tissue, Kuhl et al. found a supplemental cancer detection rate of 15.5 per 1000 cases, with a median size of MRI-detected tumors of 8 mm and no interval cancers in the collective of 2120 women with an observation period of 7007 women-years (68).

Based on these data, a recent cost-benefit analysis (**Table 4**) simulating screening costs only has indicated that despite higher costs in the short run, triennial MRI screening of women at average risk could be cost-saving compared to annual mammography after 6 years, assuming costs per MRI of USD 400 (69).

**Table 4:** Economic evaluation on MRI-based breast cancer screening of women at average risk.

Study	Study population	Economic model; perspective	Comparators	Cost of MRI	Outcome measures	WTP-threshold
Mango et al. (69)	Women at average risk	Monte Carlo simulation model (cost-benefit analysis), US healthcare system	Triennial breast MRI, annual conventional mammography	USD 550 (2019)	Screening costs in USD	n/a

n/a, Not applicable.

## Discussion

Indications of MRI-based screening have gradually been extended over the last 20 years (**Table 1**) along with increasing evidence on improved cancer detection rates of breast MRI in different risk groups (40, 41, 45–47, 70–72).

With evidence on a high specificity of breast MRI in expert hands (12–14), as well as evidence against adverse effects when using repetitive macrocyclic contrast media-enhanced breast MRI for screening (73), financial concerns represent the main obstacle to an increased application of breast MRI in screening women beyond the subgroup of women at high risk. Along with increasing evidence on the safety and efficacy of MRI-based breast cancer screening in women at intermediate risk, the technique has demonstrated to be cost-effective in a variety of indications and conditions that have not yet been implemented in population screening programs. Randomized controlled studies on MRI-based breast cancer screening in women at average risk are unavailable so far.

Major determinants of cost-effectiveness in screening of various risk groups using breast MRI have been identified, with examination costs being identified as the most potent driver of cost-effectiveness. Diagnostic performance, incidence and prevalence rates could be identified as major determinants as well. However, due to heterogeneity of the modeling approaches, they often cannot be quantitatively compared.

### Impact of diagnostic performance

While early studies on the diagnostic performance of breast MRI in high-risk screening have indicated lower levels of sensitivity and specificity of 46 - 77% and 81 - 95%, respectively (74–76), the most recent prospective multi-center trials confirmed a largely superior sensitivity of 90 - 93% and a specificity of 89 - 98% (15–17). These shifts in diagnostic performance may be attributable to premature technique as well as initially limited experience with the new technique. At the same time, examination costs have gradually declined over the previous years. Therefore, initial economic evaluations need to be interpreted in the light of their input parameters and assumptions.

While excellent sensitivity is considered a prerequisite for effective screening, generally accomplished by breast MRI (15–17), specificity has been identified as a major determinant for the economic success of MRI screening. This has particular importance for breast cancer screening as positive findings often result in invasive procedures such as biopsies and surgeries, often associated with psychological burden and significant costs.

Quality assurance, benchmarking and performance metrics should be monitored when designing future cost-effective screening programs, since optimal specificity relies on high-quality imaging and image interpretation (77, 78). Multicentric evaluation has shown that different decision algorithms, such as the Kaiser score, can substantially help to improve the specificity of breast MRI (79–81) and compensate for reader experience to some degree (82).

### Facets of economic evaluations

From an economical point of view, the costs of setting up a screening program and performing the first screening rounds (prevalence rounds) are initially higher, but decrease over time as initial expenses include training, quality assurance and supervision. The prevalence rounds are known to yield more false positive findings, i.e. more recommendations for biopsies as the stability of equivocal lesions cannot be determined without prior imaging.

In subsequent screening rounds, specificity therefore increases and less false positives are observed (15, 19). As a consequence, the costs of MRI-based screening are higher for women entering screening programs. To capture all economic effects of screening, the stage and nodal status of MRI-detected cancers need to be considered in economic modeling as well as reduced costs for treatment and long-term follow-up. Comprehensive economic evaluations need to account for these short- and long-term effects in order to yield valid conclusions.

Other factors that improve cost-effectiveness of breast MRI are high prevalence and incidence, i.e. higher risk of breast cancer, that can be influenced by further refining the screening population by more sophisticated risk models.

### Overdiagnosis

Overdiagnosis refers to the detection of clinically insignificant breast cancer that does not have an impact on a woman’s life expectancy. Concerns on overdiagnosis have been raised after the incidence of invasive breast cancer and ductal carcinoma *in situ* (DCIS) increased particularly in early mammography screening rounds (9, 83). As the significance of cancer currently cannot be distinguished with any reliability by histology and cannot be identified on an individual level, a number of women potentially receive unnecessary work-up and therapy. However, when adjusting for breast cancer risk and lead time, most plausible estimates of overdiagnosis due to screening mammography range between 1% to 10% (84, 85).

At the same time, underdiagnosis represents a major challenge and many women are underserved by conventional screening as evidenced by breast cancer morbidity and mortality statistics. Underdiagnosis hereby is defined as not detecting a present cancer, i.e. false-negative finding. Notably, MRI preferentially detects more aggressive tumors (22) and may be used to predict the course of disease (86), thereby providing an angle for exploring strategies to escalate or de-escalate treatment.

Modeling overdiagnosis remains a challenge in economic evaluations as evidence on breast MRI screening is limited and accurate numbers are scarce. Several microsimulation studies have incorporated estimates of overdiagnosis (59, 64).

### Extrapolation from real-world data

Model-based economic evaluations provide valuable insights by simulating long-term costs and outcomes and by modeling different screening strategies for the purpose of decision analysis. However, the more economic models rely on extrapolations from real-world data, the more the validity of the findings may be limited. The results should therefore be interpreted with caution.

For instance, when various screening intervals are simulated in economic modeling, the outcomes are not directly based on empirical evidence. Economic models should be designed to rely on real-world scientific evidence as much as possible, in order for the results to not represent artificial interrelations depending on the modeling approach. For instance, the longer the screening interval, the lower the costs of screening. If increased interval cancer rates and advanced disease stages of belated diagnoses are not properly accounted for, the resulting ICER may be artificially low for prolonged screening intervals. This could potentially result in an endorsement of longer screening intervals that is not directly based on empirical outcomes, which is why a cautious interpretation is advised (87). Further, prolonged screening intervals may affect attendance rates of screening and women’s’ psychological comfort, which might result in unforeseen but relevant economic effects. Therefore, recommendations on the length of screening intervals should not be derived from economic simulations alone.

Along with the development of breast MRI as a screening technique, the methodology of economic evaluations has evolved as well. While early cost-effectiveness analyses applied various techniques such as Monte Carlo simulations based on spreadsheet programs and statistics software, dedicated software for economic modeling has become state of the art for contemporary cost-effectiveness analyses. Markov Modeling has been established as a robust economic approach in contrast to Microsimulation models (e.g. MISCAN) that have a strength in accurately modeling epidemiological contexts.

### Note on abbreviation

Examination costs depend on various healthcare policy factors, including reimbursement schemes and organization as well as the funding of a screening program. Since small cost reductions have a significant impact on the cost-effectiveness of a technique, there have been many attempts to streamline workflows, reduce non-value added time, and to reduce acquisition and image reading times of breast MRI.

Abbreviated breast MRI, i.e. restricting the number of sequences in breast MRI to an essential “abbreviated” limit, has been proposed as a means to reduce the costs of MR-based screening. Although initially defined as solely pre- and post-contrast sequences with subtracted and maximum-intensity projection images (71, 72), a variety of abbreviated protocols have recently been proposed in clinical studies (73–76) that reported varying levels of specificity. So far, however, a standard definition of abbreviated protocols has not been achieved, resulting in a heterogeneous diagnostic landscape, highly individual abbreviation approaches (17, 77) and - consecutively - in varying results regarding economic potential and implications.

While, likely due to small study and patient selection bias, a similar diagnostic performance compared to full diagnostic protocols was reported in the majority of retrospective studies (88), abbreviated breast MRI suffered from reduced specificity in the most recent prospective multi-center trial (20). High-level evidence on the diagnostic performance of different degrees of protocol abbreviation remains scarce. This is why caution is advised when implementing abbreviated protocols.

So far, the cost of abbreviated breast MRI has not been assigned a fixed reimbursement. First experiences of implementing abbreviated breast MRI as a self-played, supplemental screening tool in the U.S. have shown that three examinations per hour may be considered feasible instead of one full diagnostic protocol, with a scan time of less than 10 minutes at USD 250 per examination (89).

At the same time, innovative techniques such as parallel imaging and deep learning-based reconstruction algorithms (90, 91) have reduced examination times of full diagnostic protocols that effectively overlap with the definition of AB-MRI without a detrimental effect on diagnostic performance. For example, a full diagnostic protocol at our institution including T2w imaging, DWI and dynamic contrast enhanced sequences with pre- and 5 post-injection series is acquired in less than 10 minutes (Magnetom Sola, 16 channel coil, Siemens Healthineers), which meets the most common requirements of an abbreviated protocol in terms of acquisition time, yet offers access to the full diagnostic accuracy of breast MRI.

### Limitations of economic modeling

Economic evaluations are afflicted with well-known methodological constraints. The technique is based on a utilitarian approach (92). Large effects and benefits are valued more than smaller effects regardless of the affected patients and the actual needs of those patients. For instance, treatment-related health outcomes in young patients with mild chronic conditions may be substantially larger than health outcomes of oncologic patients in end-of-life conditions which may raise ethical concerns on equity and fairness.

Model-based analyses rely on a simplification of complex clinical pathways and heterogenous patient groups that have to be translated into economical models. In reality, adherence to screening recommendations varies and women enter screening programs at different points in time, skip or prolong screening intervals and deviate from therapeutic and diagnostic pathways projected in economic models.

The validity of the modeled costs and effects depends on the quality of input data. However, as high-quality data on costs and outcomes are scarce, applicability to different contexts is limited. Many cost-effectiveness analyses lack calibration of input parameters and external validation and are therefore prone to bias (93).

## Conclusion

With increasing evidence on the efficacy and safety of MRI-based breast cancer screening, available cost-effectiveness analyses indicate a strongly favorable economic value compared to conventional screening for a variety of risk groups.

MRI screening is expected to be extended from women with high risk of breast cancer towards women with dense breast tissue. Cost-effectiveness of breast MRI screening in women with dense breast tissue could be demonstrated based on the most recent evidence from prospective multi-center trials. However, further studies are necessary to evaluate the outcomes and cost-effectiveness of screening women at average risk.

## Author contributions

FT: Conceptualization, Investigation, Methodology, Visualization, Writing – original draft, Writing – review & editing. PB: Supervision, Writing – review & editing. MF: Methodology, Supervision, Writing – review & editing. CK: Conceptualization, Investigation, Supervision, Validation, Writing – original draft, Writing – review & editing.
